# WORKING MEMORY MODERATES DEPRESSIVE SYMPTOMS AFTER PARTNER MORTALITY: HEALTH AND RETIREMENT STUDY

**DOI:** 10.1093/geroni/igac059.1391

**Published:** 2022-12-20

**Authors:** David Brush, Daniel Paulson, Robert Dvorak

**Affiliations:** University of Michigan, Ann Arbor, Michigan, United States; University of Central Florida, Maitland, Florida, United States; University of Central Florida, Orlando, Florida, United States

## Abstract

Grief is conceptualized as a process after which the individual becomes better adapted to changed living conditions after a loss. The Selection, Optimization, and Compensation with Emotion Regulation (SOC-ER) model posits that working memory may be necessary for mitigation and resolution of grief. This study examined the hypothesis that working memory facilitates successful grieving following the loss of an intimate partner. Participants include 3,599 respondents of the longitudinal Health and Retirement Study (HRS) who experienced spousal mortality between 1994 and 2014. Working memory was measured assessed using Serial 7’s, and depressive symptoms were assessed using the 8-question CES-D. Latent-growth models were used to estimate rate of change in depressive symptoms up to loss-of-spouse (baseline event), and then subsequent to that loss. Missing data were handled using full-information maximum likelihood. Sample participants had an average age 78.04 (SD = 7.32) at the time of their spouse’s death and were disproportionately female (69.10%), White/Caucasian (82.30%), non-Hispanic (92.37%), and completed an average of 11.61 (SD = 3.42) years of education. The hypothesized level 2 model fit the data very well: χ2(56)=61.323, p=.29 RMSEA=0.005 [0.000 0.012]; CFI=0.998, SRMR=0.028. Initial depressive symptom endorsement was significantly related to working memory ability. Working memory also moderated the relationship between depressive symptom endorsement and time, where individuals with better working memory tended to report lower depressive symptoms and demonstrated a lesser increase in depressive symptoms. In conclusion, working memory may be one determinant of successful bereavement. Findings support application of the SOC-ER model to the study of grief.

